# The Effect of Different Mixing Methods on the pH and Solubility of Mineral Trioxide Aggregate and Calcium-Enriched Mixture

**Published:** 2015-03-18

**Authors:** Shahriar Shahi, Negin Ghasemi, Saeed Rahimi, Hamid Reza Yavari, Mohammad Samiei, Maryam Janani, Mahmood Bahari

**Affiliations:** a *Dental and Periodontal Research Center, Dental Faculty, Tabriz University of Medical Sciences, Tabriz, Iran; *; b * Department of Endodontics, Dental Faculty, Tabriz University of Medical Sciences, Tabriz, Iran; *; c *Department of Restorative Dentistry, Dental Faculty, Tabriz University of Medical Sciences, Tabriz, Iran*

**Keywords:** Calcium-Enriched Mixture, CEM Cement, Mineral Trioxide Aggregate, MTA, pH, Solubility

## Abstract

**Introduction:** The aim of this study was to evaluate the effect of different mixing techniques on the pH and solubility of mineral trioxide aggregate (MTA) and calcium-enriched mixture (CEM). **Methods and Materials:** Five samples were prepared from each biomaterial with different mixing techniques including hand-, amalgamator- or ultrasonic-mixing and were then placed in pre-weighted plastic tubes to determine their pH values. Each tube was then incubated in 10 mL deionized distilled water for 1 h at 37^º^C. An electrode was placed in the fluid in each flask at 24^º^C and the pH was recorded. In the next stage, six samples from each mixing technique/material were separately placed in glass bottles containing 50 mL of distilled water at 37^º^C for 1 h and were let dry for 1 h at 37^º^C. The samples’ weights were measured and recorded twice. The procedure was repeated at 1-, 7- and 21-day intervals. Data were analyzed with the repeated measures ANOVA (for solubility) and two-way ANOVA (for pH) and then the post-hoc Tukey’s test was done.** Results:** The pH of the materials was not significantly affected by mixing methods. (*P*=0.8 for CEM and *P*=0.1 for MTA). The solubility of all test groups was within the acceptable range (=3%). However, the solubility of CEM at 1- and 21-day intervals was significantly different (*P*=0.03 for 1 day and *P*=0.001 for 21 days). Different mixing techniques had significant effects on the solubility of MTA at the three time points (*P*=0.004, 0.003 and 0.002 for 1-, 7- and 21-day intervals, respectively). **Conclusion:** The pH of biomaterials was not influenced by the mixing technique and their solubility was within the acceptable range.

## Introduction

The solubility of calcium phosphate cements and more specifically biomaterials such as mineral trioxide aggregate (MTA) and calcium-enriched mixture (CEM) cement has always attracted a lot of attention [1-3]. The ultimately important mechanism of osteo/dentino/cementogenic induction by MTA, is attributable to various factors including its profound sealing and alkalinity [2]. On the other hand, the excellent biocompatibility of MTA is attributed to its alkaline pH and the potential to release calcium ions [4]. An alkaline pH is important for hard tissue induction and antimicrobial activity considering that resistant endodontic bacteria such as *Enterococcus faecalis*, are destroyed at a pH values over 11 [5, 6].

The physical and chemical properties of dental materials might be influenced by the mixing technique. Mechanical trituration can decrease air-filled spaces between the material particles and increase the odds of wetting of particle surfaces thus leading to improved uniformity of the mixture [6-8]. Ultrasonic energy, as a mixing technique, influences the dispersion of particles arranged in clusters next to each other. Therefore, the overall reactive surface area increases. Based on the results of previous studies, ultrasonic energy can increase the compressive and tensile strengths and the density of the materials and decrease setting time, which finally improve handling properties [6].

MTA is a mixture of three powders: Portland cement, bismuth oxide and gypsum [9-11]. MTA is used as a root-end filling material, for sealing the perforations and in apexification procedures due to its excellent biocompatibility and hard tissue inducing potential [12-15]. 

On the other hand the main components of CEM cement include metallic oxides and hydroxides, calcium phosphate and calcium silicate [12, 16]. The clinical applications of CEMcement are similar to those of MTA; however, it does not have the disadvantages of MTA, such as long setting time and difficult handling. In addition, its flow and film thickness are better than MTA [17]. Similar to MTA, the main ingredient of CEM cement is calcium (27 wt% in MTA and 51.81 wt% in CEM) [17]. Hydration reactions during mixing, results in the release of calcium hydroxide which is converted to calcium and hydroxyl ions in an aqueous environment that leads to an increase in the pH value [18]. Studies have shown that CEM cement has an alkaline pH (10.71) similar to that of MTA (10.61) [17]. 

Since there are no published reports available on the effects of different mixing techniques on solubility and pH of MTA and CEM cement, the present study was designed to evaluate the effect of different mixing techniques on the mentioned physical properties of these biomaterials.

## Materials and Methods

***Preparation of samples***

Before starting, the mixing pads, spatulas and glass slabs were placed at 23±1^º^C for 1 h. The powders and liquids were mixed using conventional, amalgamator or ultrasonic techniques. In the conventional technique, the liquid and powder were hand-mixed based on the manufacturer’s instructions. In the ultrasonic technique, mixing of the powder and liquid was exactly the same except that the procedure was carried out by the tip of an ultrasonic scaling device (Juya Electronics, Iran). In the amalgamator technique, proper ratios of the liquid and powder were placed in the mixing chamber of an amalgamator (Duomat II, Dental und Goldhalbzeug, 600 Frankfurt, Germany) and triturated for 20 sec. 

***Determination of pH***

A Metrohm 744 pH meter (Metrohm Ltd, Herisa, Switzerland) was used to determine the pH values. The device was tested before the experiment using standard solutions with pH vales of 4 and 7. The mixed cements were placed in plastic tubes measuring 10 mm in length and 1.5 mm in diameter. The tubes were weighed accurately before and after being filled with cement samples. Five samples were prepared from each cement with each mixing technique. Each tube was separately placed in a flask containing 10 mL of deionized distilled water for 1 h at 37ºC. Then an electrode was placed in the liquid-containing flask at 24ºC and the pH value was recorded***.***

**Table 1:** Mean (SD) of weight loss (solubility) in μg

**Mixing technique**		**Mean (SD) of weight loss (μg) **		
		**1 day **	**7 days**	**21 days**
**Manual**	**CEM **	0.09 (0.09)	0.09 (0.01)	0.09 (0.01)
	**MTA**	0.18 (0.04)	0.15 (0.02)	0.14 (0.01)
**Amalgamator**	**CEM **	0.12 (0.01)	0.11 (0.08)	0.11 (0.03)
	**MTA**	0.26 (0.02)	0.23 (0.02)	0.22 (0.02)
**Ultrasonic**	**CEM **	0.09 (0.03)	0.08 (0.07)	0.08 (0.07)
	**MTA**	0.25 (0.01)	0.22 (0.01)	0.21 (0.01)

***Determination of solubility***

Solubility was determined based on the modified ADA guidelines No.30. According to ISO 6876 and ADA protocol, the solubility of samples were measured in DW and the solubility of ≤3% was considered acceptable [19]. The materials under study were mixed using the methods described above and were placed within the disk-shaped molds measuring 20×1.5 mm. The mixing and weighing of the samples were carried out by one operator at 23±2^º^C and a relative humidity of 50±5%. Six samples were prepared for each mixing technique from each material. Then the samples were stored for 21 h at 100% relative humidity. In the next stage, each sample was separately placed in glass bottles containing 50 mL of distilled water at 37^º^C for 1 h. Subsequently, all the samples were left to dry at 37^º^C for 1 h and were then weighed. After weighting, the samples were returned to the same bottles without changing their water content. The drying and weighing steps of the samples were reported at 1-, 7- and 21-day intervals by subtracting W2 (the weight of sample at the end of related time interval) from W1 (the initial weight) indicating the weight loss. The amount of weight loss in μg was interpreted as solubility. The percentage of solubility was also calculated using the following formula: (weight loss×100)/W1.

***Data analysis***

Data were analyzed with repeated measures ANOVA (for solubility) and two-way ANOVA (for pH). The post-hoc Tukey’s test was used for two-by-two comparison of the groups. SPSS software (SPSS version 18.0, SPSS, Chicago, IL, USA) was used for the analysis of data. The level of significance was set at 0.05.

## Results

***Solubility***

With all mixing techniques and at all three time intervals, MTA exhibited the highest solubility values. Evaluation of solubility at 1- and 21-day intervals with the three different mixing techniques revealed significant differences (*P*=0.001 and 0.006, respectively). Two-by-two comparisons of the subgroups showed that the solubility at above-mentioned intervals was significantly higher with the amalgamator mixing than the two other techniques. However, there were no significant differences between hand and ultrasonic techniques. There were no significant differences in the solubility of CEM cement between three different mixing techniques at 7-day interval (*P*=0.09). Mixing technique had a significant effect on solubility of MTA between the three time intervals (*P*=0.004, 0.003 and 0.002 for 1-, 7- and 21-day intervals), with significantly lower solubility with hand-mixing technique. However, there were no significant differences between the two other techniques. The difference between two materials was significant except in 7- and 21-day intervals in manual-mixing method (*P*>0.05). 

**Table 2:** Mean (SD) of pH values

**Mixing technique**		**Mean (SD) of pH **
**Manual**	CEM	10.86 (0.12)
	MTA	10.9 (0.08)
**Amalgamator**	CEM	10.76 (0.05)
	MTA	11.11 (0.11)
**Ultrasonic**	CEM	10.57 (0.10)
	MTA	11.07 (0.06)

The percentage of weight loss in all groups was below 3% which indicates that the solubility of all test groups was within the acceptable range (=3%) according to modified ADA guidelines [19]. However, the highest amount was reported for 1-day MTA samples mixed with amalgamator (0.0026%). 

***pH***

The pH of MTA was higher than CEM cement with all three mixing techniques. The pH of both biomaterials was not significantly affected by different mixing techniques (*P*=0.8 and 0.1, respectively). The difference was not significant in any mixing methods (*P*>0.05) (Table 2).

## Discussion

In the present study, the effect of three different mixing techniques (hand-, amalgamator- and ultrasonic-mixing) on two properties (pH and solubility) of CEM cement and MTA was evaluated. The pH was not affected by the mixing technique in any of the materials. Regarding the solubility, CEM cement exhibited a higher solubility with the amalgamator technique at 1-day and 21-day intervals. The solubility of MTA was influenced by the mixing technique at all the three time intervals, with the lowest weight loss (solubility) belonging to hand-mixed samples. 

In order to achieve optimal properties with hydraulic cements, the powder particles should be thoroughly mixed with liquid [7]. The mixing technique forms a foundation for an effective contact between powder particles and the liquid to achieve optimal physicochemical and biologic properties. Based on the results of a study by Basturk *et al.* [6] ultrasonic vibration results in higher surface microhardness compared to the manual technique. 

Capabilities such as a proper seal in the presence of blood and moisture, biocompatibility and hard tissue induction potential are important properties of materials such as MTA and CEM cement, which are useful for purposes such as filling the root-end cavities, root canal treatment of immature teeth and pulp capping [12, 20, 21].

Calcium is released during the setting process of MTA in the form of hydroxide which is considered the most important chemical component and considering the presence of large amounts of calcium in the structure of CEM cement, a similar setting reaction is expected [4, 17, 22]. Set MTA preserves its high pH value for a long time and can release its soluble component into the environment at a decreasing rate for 78 days [23].The pH of freshly mixed MTA is 10.2 and reaches 12.5 after 3 h, which is higher than that of Portland cement based on the results of previous studies. CEM cement also has high alkalinity which makes it ideal regarding bactericidal activity, proper sealing and biocompatibility [12]. The results of the present study showed that the pH of CEM is comparable to that of MTA, consistent with the results of the study by Asgary *et al.* [17]. 

Solubility and disintegration are directly related to the material’s sealing ability because these two properties are responsible for the preservation of the material’s dimensional stability [2]. The silica matrix is the insoluble component of MTA and preserves its integrity in an aqueous environment. Therefore, there is no concern about complete dissolution of MTA [4]. In fact, dissolution of similar hydraulic MTA cements is not a reason to contraindicate their use in the clinic because dissolution is an important biologic process involved in the release of hydroxyl ions into the environment, which results in an alkaline pH that ultimately promotes regeneration process and antimicrobial activity [4, 24]. 

It can be concluded that regarding solubility, it is important for CEM cement to be mixed in an amalgamator rather than the two other techniques; but solubility of the two other mixing techniques become similar to that of amalgamator-mixing with the pass of time. In other words, as the time goes by, the solubility of ultrasonic-mixed CEM cement approaches that of CEM samples mixed with amalgamator. Regarding the solubility of MTA after one and 7 days, the solubility was lower with the hand-mixing method compared to the two other mixing techniques. In order for the MTA to preserve its solubility under similar conditions, hand-mixing is advisable.

Regarding the pH value it can be concluded that the three different mixing techniques did not result in significant differences in pH values. In other words, the mixing technique had no effect on the pH of the final set material. A high pH value has a direct relationship with the material’s antibacterial activity, which is important for clinical applications. In the present study, the pH values of MTA were higher than those of CEM cement, contrary to the results reported b Asgary *et al.* [17]. The difference in pH values between the two studies might be attributed to differences in time intervals at which pH values were measured.

## Conclusion

Under the limitation of this study, it can be concluded that MTA and CEM cement had acceptable solubility; in addition, pH of the biomaterials were not influenced by the mixing techniques.
